# Immune‐based transcriptomic signature predicts CDK4/6 inhibitor efficacy in HR+/HER2– breast cancer

**DOI:** 10.1002/ctm2.70426

**Published:** 2025-08-07

**Authors:** Eudald Felip, Edurne Garcia‐Vidal, Sara Cabrero‐de las Heras, Adrià Bernat‐Peguera, Beatriz Cirauqui, Milana Bergamino, Vanesa Quiroga, Iris Teruel, Angelica Ferrando‐Díez, Anna Pous, Assumpció Lopez‐Paradís, Laia Boronat, Marga Romeo, Ricard Mesía, Pedro Luis Fernandez, Bonaventura Clotet, Eva Riveira‐Muñoz, Anna Martínez‐Cardús, Ester Ballana, Mireia Margelí

**Affiliations:** ^1^ Institut Català d'Oncologia‐Badalona Hospital Universitari Germans Trias i Pujol Barcelona Spain; ^2^ Translational Program in Cancer Research (CARE) Program in Health Research Institute Germans Trias i Pujol Hospital Universitari Germans Trias i Pujol, Universitat Autònoma de Barcelona Barcelona Spain; ^3^ IrsiCaixa Barcelona Spain; ^4^ Badalona‐Applied Research Group in Oncology, Germans Trias i Pujol Barcelona Spain; ^5^ Institute for Bioengineering of Catalonia Barcelona Spain; ^6^ Department of Pathology Hospital Germans Trias i Pujol Barcelona Spain; ^7^ Centro de Investigación Biomédica en Red de Enfermedades Infecciosas, CIBERINFEC Madrid Spain

1

Dear Editor,

Cyclin‐dependent kinase 4/6 inhibitors (CDK4/6i) have revolutionised the management of hormone receptor‐positive (HR+), HER2‐negative (HER2‒) advanced breast cancer (ABC).^1^, ^2^ However, the molecular mechanisms driving tumour resistance and subsequent therapies remain a challenge.^3^ Here, we identified and validated a novel transcriptomic immune‐based signature (‘Key Immune Activation’ [KIMA] signature) that predicts efficacy to CDK4/6i in HR+/HER2– ABC. KIMA signature provides a clinically actionable biomarker that has the potential to guide immune‐based combination strategies and personalised treatment in ABC patients.

We conducted a prospective observational study of 100 HR+/HER2– ABC patients who initiated CDK4/6i therapy in combination with endocrine therapy between March 2018 and April 2022 at the Institut Català d'Oncologia, Badalona. Among them, 91 patients had sufficient clinical follow‐up for efficacy evaluation. Pre‐treatment tumour samples were available for 55 patients (Figure 1A). Clinical characteristics and intrinsic subtypes were characterised (Table 1). Overall, the clinical characterisation of the cohort revealed a median progression‐free survival (PFS) of 14 months (interquartile range [IQR]: 5.6–26.6) and a median overall survival (OS) of 42.8 months (IQR: 20.3–47.3; Figure S1A). As expected, the line of treatment had the most significant impact on PFS, with first‐line patients showing better outcomes (first line: 30.4 months vs. second line or later lines: 9.3 months, *p* = .001). Other clinical factors, such as endocrine resistance and hepatic metastasis, showed consistent trends towards shorter PFS, aligning with results from prior larger studies^4^ (Figure S1B). Intrinsic subtype^5^ analysis by prediction analysis of microarray 50 (PAM50) revealed a predominance of luminal tumours, with an increased proportion of HER2‐enriched and basal subtypes in metastatic lesions compared to primary tumours (*p* = .034; Figure S2A). Among first‐line‐treated patients, luminal‐subtype tumours tended towards longer PFS (Figure S2B).

**FIGURE 1:**
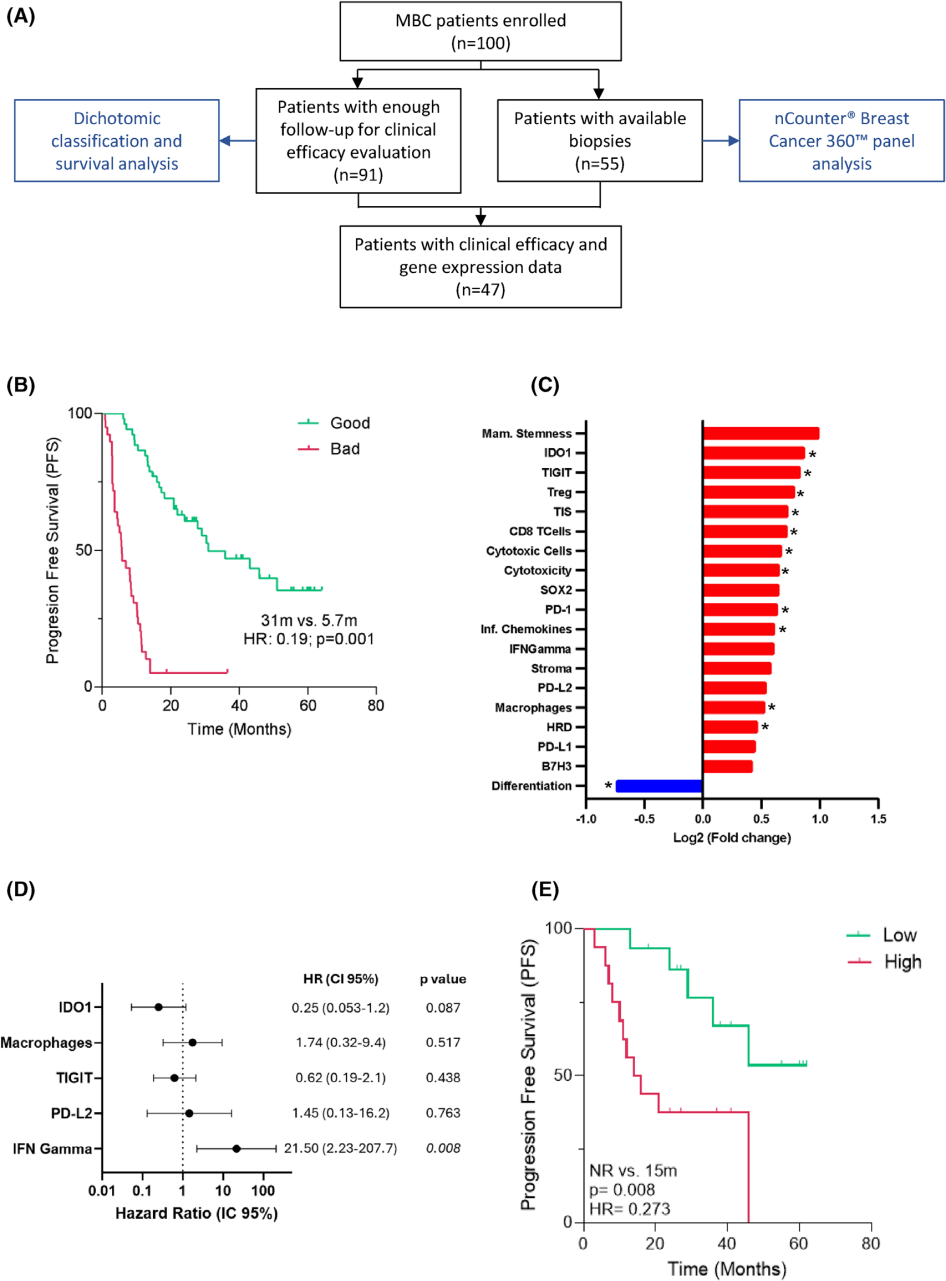
Cohort characteristics and the influence of immune‐based Breast Cancer 360™ (BC360™) gene signatures on the efficacy of cyclin‐dependent kinase 4/6 inhibitor (CDK4/6i) treatment. (A) Flowchart detailing the study plan. Among all participants enrolled, 91 patients had sufficient follow‐up to evaluate the clinical efficacy, and the transcriptomic analysis in tissue biopsies was performed in 55 of them. (B) Kaplan‒Meier analysis of progression‐free survival (PFS) in good and bad efficacy groups treated with CDK4/6i, according to the clinical classification developed. (C) Top differentially enriched BC360™ signatures (*p* < .1) in the bad efficacy group compared to the good efficacy group in the entire cohort (*n* = 47), based on Log2 gene expression (Log2FC). Significantly downregulated or upregulated signatures are highlighted in blue or red, respectively, and significant differences (*p* < .05) are denoted by an asterisk (*). (D) Multivariate survival analysis of the five relevant BC360™ signatures in univariate analysis (*p* < .1, Table S4) in first‐line patients (*n* = 31). (E) Kaplan‒Meier analysis describing the impact of the BC360™ IFN‐γ signature on patients PFS, stratified by high versus low expression (cut‐off based on the optimal threshold calculated from the receiver operating characteristic [ROC] curve). *p*‐Value and hazard ratio (HR) determination were calculated using the log‐rank test. CI, confidence interval.

**TABLE 1:** Clinical characteristics of the study cohort.

	Cohort (*n* = 100)	Efficacy group		
		Good (*n* = 52)	Bad (*n* = 39)	*p*‐Value^a^
Age, median [IQR]	62.3 [50.6–69.8]	60.45 [44.28–80.62]	58.52 [39.2–77.12]	.270
CDK4/6i, *n* (%)				.199
Abemaciclib	19 (19%)	9 (17.30%)	7 (17.95%)	
Palbociclib	60 (60%)	29 (55.77%)	27 (69.23%)	
Ribociclib	21 (21%)	14 (26.93%)	5 (12.82%)	
Line, *n* (%)				.985
First M1 line	60 (60%)	32 (61.54%)	22 (56.41%)	
Second line	13 (13%)	6 (11.54%)	4 (10.26%)	
Third or other line	27 (27%)	14 (26.92%)	13 (33.33%)	
Endocrine therapy, *n* (%)				.329
Tamoxife	9 (9%)	5 (9.62%)	3 (7.69%)	
Fulvestrant	53 (53%)	16 (30.77%)	18 (46.15%)	
AI	27 (27%)	31 (59.62%)	18 (46.15%)	
GhRh analeg, *n* (%)				.852
Yes	23 (23%)	10 (19.23%)	9 (23.07%)	
No	77 (77%)	42 (80.77%)	30 (76.92%)	
M1 de novo, *n* (%)	29 (29%)	16 (30.77%)	9 (23.07%)	.564
Metastatic location, *n* (%)				.980
Visceral	44 (44%)	22 (42.61%)	20 (51.28%)	.523
Hepatic	26 (26%)	12 (23.08%)	14 (35.90%)	.269
Only Bone	27 (27%)	17 (32.69%)	7 (17.95%)	.180
CNS	4 (4%)	2 (3.84%)	1 (2.56%)	.999
PAM50 subtype, *n* (%)	55/100	28/52	19/39	.361
Luminal A	21 (38.19%)	11 (39.28%)	10 (52.63%)	
Luminal B	26 (47.27%)	12 (42.85%)	9 (47.37%)	
HER2‐E	6 (10.90%)	3 (10.71%)	0 (0%)	
Basal‐Like	2 (3.64%)	2 (7.14%)	0 (0%)	
N/A	45	24	20	
CDK4/6i status, *n* (%)				*.001*
Stop due PD	32 (32%)	28 (53.84%)	2 (5.13%)	
Stop due toxicity	8 (8%)	4 (7.77%)	0 (0%)	
Ongoing	60 (60%)	20 (38.46%)	37 (94.87%)	
Last control, *n* (%)				*.001*
Alive	51 (50%)	37 (71.15%)	8 (20.51%)	
Death due to PD	47 (47%)	15 (28.84%)	30 (76.92%)	
Death due other causes	2 (2%)	0 (0%)	1 (2.56%)	

*Note*: Data from all patients and the two efficacy groups is shown.

Abbreviations: AI, aromatase inhibitor; CDK4/6i, cyclin‐dependent kinase 4/6 inhibitor; IQR, interquartile range; M1, metastatic; *n*, number; PD, progression disease.

^a^ *p*‐Value: Wilcoxon test for numeric variables. Fisher's test for categorical values considered significant less than  .05 (marked in italics).

Due to clinical heterogeneity, patients were stratified into two efficacy groups (good or bad) based on predefined clinical criteria derived from PFS benchmarks reported in pivotal CDK4/6i trials. Specifically, patients with hormone‐sensitive disease were categorised as good efficacy if PFS was ≥24 months, while those with hormone‐resistant disease or prior therapies were categorised using lower PFS thresholds (≥12 or ≥7 months) according to clinical context (see section Material and Methods in the Supporting Information and Figure S3). Using this approach, 57% of patients were classified in the good efficacy group (median PFS 31 months) and 43% in the bad efficacy group (median PFS 5.7 months; *p* < .001; Figure 1B). Baseline clinical characteristics were comparable between groups, supporting the need for molecular biomarkers of efficacy (Table 1). To identify potential tumour prognostic and predictive factors influencing the efficacy of CDK4/6i, we performed a transcriptomic analysis using the NanoString Breast Cancer 360™ (BC360™) panel in patients with matched tumour tissue and clinical data (Figure 1A and Table S1). We compared BC360™ predefined gene signatures between good and bad efficacy groups and identified 19 differentially expressed signatures, mostly enriched in the bad efficacy group (18/19, Figure 1C). Among the overexpressed signatures, we identified substantial enrichment in immune‐related pathways, including IDO1 (Indoleamine 2,3‐dioxygenase 1), TIGIT (T cell immunoreceptor with Ig and ITIM domains), Treg (Regulatory T cell), TIS (Tumor Inflammation Signature), CD8 (Cluster of differentiation 8 lymphocyte marker), PD‐1 (Programmed cell death protein 1), inflammatory chemokines, IFN‐γ (Interferon gamma), PD‐L1 (Programmed death‐ligand 1), PD‐L2 (Programmed death‐ligand 2) and macrophages. In contrast, the only downregulated signature was related to luminal differentiation. To further assess the clinical impact of these signatures while minimising potential biases from prior treatments, survival analysis was performed in patients receiving CDK4/6i as first‐line therapy (*n* = 31). Univariate Cox survival analysis showed that high expression of IFN‐γ, PD‐L2, TIGIT, IDO1 and macrophage signatures was associated with shorter PFS (Table S2). Multivariate analysis confirmed the increased expression of IFN‐γ signature as an independent predictor of poor outcome (hazard ratio = 21.5, *p* = .008; Figure 1D and Table S3). Furthermore, using the optimal threshold calculated through receiver operating characteristic (ROC) curve analysis, patients with high IFN‐γ signature expression had a significantly worse median PFS (15 months vs. not reached, *p* = .008), confirming the negative impact of high BC360™ IFN‐γ signature expression (Figure 1E).

To recognise the immune‐associated predictors of efficacy, we analysed differences in single‐gene expression, which led to the identification of 43 differentially expressed genes (DEGs). This analysis revealed a significant enrichment of immune‐related genes in bad efficacy group (Figures 2A and S4A‒C). We then selected all immune‐related DEGs that showed at least a 50% difference between efficacy groups (*n* = 14). Unsupervised consensus clustering effectively grouped 90% of patients with good treatment efficacy (*p* = .004; Figure 2B), identifying two gene sets that were found either upregulated (*n* = 9) or downregulated (*n* = 5) in patients classified as bad versus good efficacy groups. Principal component analysis further demonstrated that all nine genes contributed to the predictive capacity of the signature (Figure 2C,D).

**FIGURE 2:**
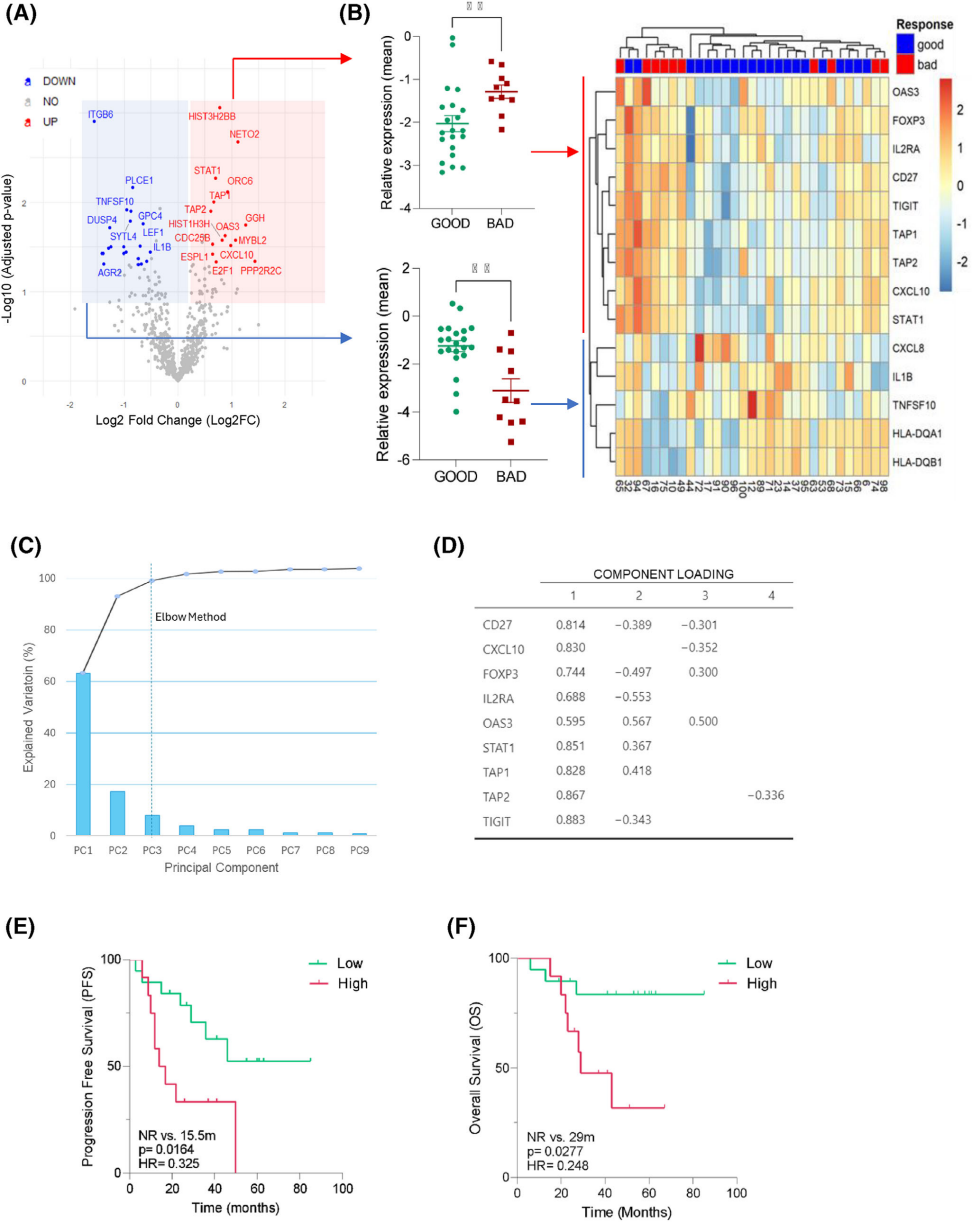
Identification of a nine‐gene immune signature that predicts efficacy of cyclin‐dependent kinase 4/6 inhibitor (CDK4/6i) treatment in first‐line patients. (A) Volcano plot of differentially expressed genes (DEGs) in the bad response group compared to the good response group in first‐line patients (*n* = 31). Significantly (*p* < .05) upregulated and downregulated genes are depicted in red and blue, respectively. (B) Scatter plots with mean ± standard deviation (SD) (right) and heatmap (left) depicting the expression levels of the 14 selected immune‐related genes in the first‐line patients (*n* = 31). Scatter plots represent the mean expression of the nine‐gene ‘Key Immune Activation’ (KIMA) (upper plot) and five‐gene downregulated gene set (lower plot) in each patient. On the right, unsupervised heatmap using hierarchical clustering with Euclidean distances for rows (genes) and columns (patients). Gene expression values were row‐scaled (*z*‐score normalisation) to highlight relative expression patterns across samples. Significance was calculated using an unpaired *t*‐test with Welch's correction. ^**^ *p* < .01. (C) Principal component analysis (PCA) of the individual genes from the KIMA signature. Scree plot of PCA of all nine genes showing the total variance explained for each component (PC). Eigenvalues (bars) and cumulative variability (line connecting dots, expressed as a percentage) are presented for all nine factors. (D) Computing of all variables on the first four PCs and their corresponding loadings. (E) Kaplan‒Meier survival curves showing progression‐free survival (PFS) and (F) overall survival (OS) of first‐line patients (*n* = 31) stratified according to high versus low expression (cut‐off based on the optimal threshold calculated from the receiver operating characteristic [ROC] curve) of the KIMA signature. The log‐rank test was used for calculating the *p*‐value and determining the hazard ratio (HR).

Survival analysis revealed that only the expression levels of the upregulated nine‐gene set correlated with differential PFS and OS, leading to the definition of the KIMA signature, which included *CXCL10*, *OAS3*, *STAT1*, *CD27*, *TIGIT*, *IL2RA*, *FOXP3*, *TAP1* and *TAP2* genes. High KIMA expression was associated with significantly shorter PFS (15 months vs. not reached, *p* = .016) and OS (29.9 months vs. not reached, *p* = .027; Figure 2E,F). In contrast, the downregulated five‐gene set (HLA‐DQA1, HLA‐DQB1, CXCL8, TNFSF10 and IL1B) did not correlate with PFS or OS in either the first‐line setting or across all treatment lines (Figure S5A‒C). The predictive value of the KIMA signature was further evaluated in all the patients, irrespective of the line of treatment with similar results, that is, patients from the poor efficacy group presented significantly higher expression levels of the KIMA signature (*p* = .0022) and shorter PFS and OS (Figure 3A‒C). Multivariate analysis, including relevant clinical factors such as line of treatment, liver metastases and visceral disease (Figure S1B), showed that only the number of line treatment and the KIMA (or IFN‐γ) signature retained a significant impact on PFS, further supporting their independent predictive value (Figures 3D and S5D). ROC curve analysis confirmed the robust predictive performance of the KIMA signature (area under the curve [AUC] = .787), showing superior accuracy compared to the BC360™ IFN‐γ signature (AUC = .728; Figure 3E).

**FIGURE 3:**
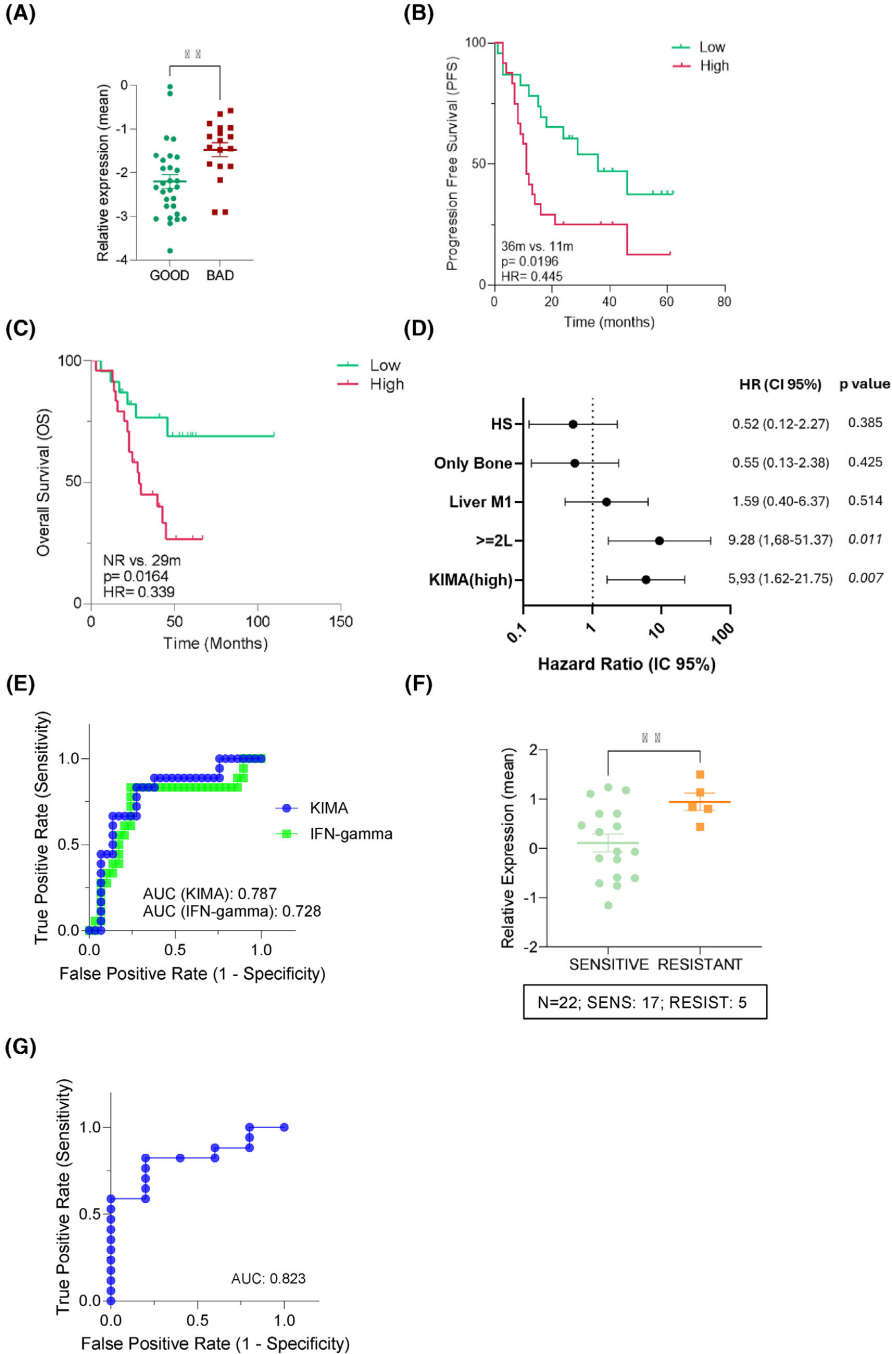
‘Key Immune Activation’ (KIMA) signature predicts efficacy in patients from all treatment lines, and its validation in the NeoPalAna cohort is also supported. (A) Scatter plot with mean ± standard deviation (SD) showing the comparison of the KIMA gene signature expression between good and bad efficacy groups, regardless of the treatment line number (*n* = 47). Significance was calculated using an unpaired *t*‐test with Welch's correction. ^**^ *p* < .01. (B and C) Kaplan‒Meier survival curves showing progression‐free survival (PFS) (B) and overall survival (OS) (C) of all patients in the cohort, stratified according to high versus low expression (cut‐off based on the optimal threshold calculated on the receiver operating characteristic [ROC] curve) of the KIMA signature. The log‐rank test was used for *p*‐value calculation and hazard ratio (HR) determination. (D) Multivariate survival analysis of the KIMA signature (high vs. low expression) adjusted for clinically relevant variables (Figure S1B), including endocrine sensitivity (Hormone‐sensitive [H‐S] vs. hormone‐resistant [H‐R]), treatment line (first‐line [1L] vs. second line or later lines [≥2L]), presence of liver metastases (Liver M1) and bone‐only disease (Only Bone) in patients (*n* = 47). (E) ROC curve and area under the curve (AUC) values illustrating the true and false favourable rates of the KIMA signature compared to Breast Cancer 360™ (BC360™) IFN‐γ signature across the entire cohort (*n* = 47). The AUC value represents the discrimination rate of the signature for the correct classification of patients. (F) Scatter plot with mean ± SD comparing the mean expression of the signature between the cyclin‐dependent kinase 4/6 inhibitor (CDK4/6i) treatment‐sensitive and resistant patient groups (*n* = 22). Significance was calculated using an unpaired *t*‐test with Welch's correction. ^**^ *p* < .01. (G) ROC curve and AUC value of the KIMA signature in the patients from the NeoPalAna cohort. CI, confidence interval.

To validate the predictive capacity of the KIMA signature in distinct clinical settings and beyond BC360™ technique, we assessed its performance in the neoadjuvant NeoPalAna study (NCT01723774).^6^ Consistent with our findings in ABC, not responder patients to neoadjuvant palbociclib displayed significantly elevated KIMA levels (*p* = .0055; Figure 3F), and the signature demonstrated strong predictive accuracy (AUC = .82 in ROC analysis; Figure 3G). While using NeoPalAna as a validation cohort can be considered a limitation due to the differences in diseases setting, it also suggests that immune‐mediated resistance mechanisms may be conserved across disease stages, supporting the robustness and broad applicability of the KIMA signature irrespective of disease scenario. Mechanistically, persistent STAT1‐driven IFN‐γ signalling characterised high‐KIMA tumours, promoting immunosuppressive microenvironments enriched in regulatory T cells (*FOXP3* and *IL2RA*), exhausted T cells (*TIGIT* and *CD27*) and immune evasion chemokines (*CXCL10*)^7^ (Figure S6). These findings underscore chronic immune activation as a key factor limiting the efficacy of CDK4/6i^8^ and support KIMA as a clinically actionable biomarker. Notably, increased immune infiltration—typically considered favourable in other subtypes—was associated with poor outcomes in ER+/HER2‒ breast cancer, suggesting distinct immunological dynamics in this context.^9^, ^10^ Our data support the hypothesis that breast cancer is immunogenic and might be targetable by immune‐modulating therapies, albeit alternative approaches to current immunotherapies directed towards the inhibition of specific immunologic signals should be considered. In this sense, the KIMA signature has multiple potential applications in clinical practice, both to identify CDK4/6i‐resistant patients harbouring elevated KIMA signature expression but also to enable the selection of combination therapies tailored to individual immune landscapes. Indeed, resistant patients could benefit from immune‐targeted interventions prior to or in combination with CDK4/6i as targeting the IFN pathway (e.g., JAK/STAT inhibitors) or immune checkpoints could help overcome this resistance.

In conclusion, the KIMA signature offers critical translational insights into immune‐mediated resistance mechanisms in HR+/HER2– ABC. By capturing the interplay between tumour‐intrinsic and immune factors, KIMA may help identify patients at risk of poor outcomes and guide personalised treatment strategies. Nevertheless, given that the KIMA signature was identified in a relatively limited cohort, prospective clinical trials are warranted to validate its predictive value and assess its generalisability. Future studies will determine how to integrate immune transcriptomic profiling into clinical practice to reduce resistance and ultimately improve therapeutic outcomes in this prevalent breast cancer subtype.

## AUTHOR CONTRIBUTIONS

Eudald Felip and Edurne Garcia‐Vidal substantially contributed to the study conception and design, methodology, patient data extraction, biological analysis, formal analysis and manuscript writing. Sara Cabrero‐de las Heras, Milana Bergamino and Adrià Bernat‐Peguera contributed to the biological data analysis and interpretation. Beatriz Cirauqui, Vanesa Quiroga, Iris Teruel, Angelica Ferrando‐Díez, Anna Pous, Assumpció López‐Paradís, Laia Boronat, Gaston Zatta and Marga Romeo contributed to the collection of clinical data, sample management and experimental procedures. Ricard Mesía, Pedro Luis Fernandez and Bonaventura Clotet contributed to study supervision, resources and critical review of the manuscript. Eva Riveira‐Muñoz and Anna Martínez‐Cardús contributed to data analysis, interpretation and manuscript revision. Ester Ballana and Mireia Margelí contributed substantially to conceptualisation, methodology planning, formal analysis, manuscript writing and critical revision, funding acquisition and overall study supervision. All authors read, reviewed and approved the final version of the manuscript.

## ETHICS STATEMENT

This research was approved by the Ethics Committee of Hospital Germans Trias i Pujol, and written informed consent was obtained from all patients prior to their enrollment in the research project.

## Supporting information



Supporting Information

Supporting Information

Supporting Information

## Data Availability

The datasets supporting this article's conclusions are included within the article (and its additional files) and available from the corresponding author upon reasonable request. Transcriptomic data and associated clinical information have been deposited in GEO (GSE265870).
