# Supplementary material for: Immune‐based transcriptomic signature predicts CDK4/6 inhibitor efficacy in HR+/HER2– breast cancer
Source: Clin Transl Med. 2025 Aug 7;15(8):e70426. doi: 10.1002/ctm2.70426 (PMC12331873; doi:10.1002/ctm2.70426)
Supplement: Supplementary file 1 — Supporting Information [file CTM2-15-e70426-s001.docx]

**Supplementary table 1.** Clinical characteristics of the subgroup of patients with available biopsy with whom the transcriptomic study has been performed.

|  | **Cohort**  **(n=100)** | **BC360™ Cohort**  **(n=55)** | **p** |
| --- | --- | --- | --- |
| **Number of line, n (%)** |  |  | 0.468 |
| 1L | 60 (60%) | 37 (67.3%) |  |
| 2L | 13 (13%) | 8 (14.5%) |  |
| >2L | 27 (27%) | 10 (18.2%) |  |
| **Hormonetherapy, n (%)** | | | 0.572 |
| Tamoxifen | 9 (9%) | 6 (10.9%) |  |
| AI | 53 (53%) | 28 (50.9%) |  |
| Fulvestrant | 27 (27%) | 21 (38.2%) |  |
| **M1 Recurrence Status, n (%)** |  |  | 0.833 |
| De novo onset | 14 (14%) | 9 (16.4%) |  |
| Recurrence >12m | 29 (29%) | 17 (30.9%) |  |
| Recurrence <12m or HT CMM | 34 (34%) | 20 (36.4%) |  |
| Chemotherapy | 23 (23%) | 9 (16.4%) |  |
| **M1 Location, n (%)** | | | 0.865 |
| Visceral | 44 (44%) | 24 (43.6% |  |
| Hepatic | 26 (26%) | 12 (21.8%) |  |
| Only bone | 27 (27%) | 16 (29.1%) |  |
| **Clinical Efficacy, n (%)** | | | 0.969 |
| Good efficacy | 42 (42%) | 23 (42%) |  |
| Poor efficacy | 39 (39%) | 21 (38%) |  |
| Not assessed | 19 (19%) | 11 (20%) |  |
| **Survival (months)** | | |  |
| PFS | 13.7m | 16m | 0.430 |
| OS | 42.8m | 48.9m | 0.770 |

*n: number , m = months, M1: metastatic,, PFS=progression free survival, OS= overall survival. ** p value. Wilcoxon_test for numeric variables. Fisher test for categorical values. Log-Rank for survival analysi. It has been considered significant less than 0.05 (marked in italics).

**Supplementary Table 2.** Differential Expression Analysis between Good and Bad Outcomes, Including all Signatures with their Corresponding Differences, and p-values. Total Sample Size: n=47 Patients (excluding those for whom outcomes cannot be reliably determined, n=55).

| **Signature** | **Bad Group mean expression** | **Good Group mean expression** | **Difference between groups** | **p value** |
| --- | --- | --- | --- | --- |
| **CD8_T_Cells** | **6.070** | **5.345** | **0.726** | ***0.010*** |
| **Differentiation** | **7.592** | **8.336** | **-0.744** | ***0.012*** |
| **TIGIT** | **5.439** | **4.602** | **0.837** | ***0.013*** |
| **TIS** | **7.021** | **6.289** | **0.732** | ***0.013*** |
| **Treg** | **4.026** | **3.237** | **0.789** | ***0.018*** |
| **IDO1** | **6.253** | **5.377** | **0.876** | ***0.019*** |
| **Cytotoxic Cells** | **4.544** | **3.866** | **0.678** | ***0.020*** |
| **Cytotoxicity** | **4.317** | **3.657** | **0.659** | ***0.022*** |
| **HRD** | **4.359** | **3.885** | **0.474** | ***0.023*** |
| **Macrophages** | **6.768** | **6.230** | **0.538** | ***0.024*** |
| **PD-1** | **4.432** | **3.788** | **0.644** | ***0.031*** |
| **Inflammatory-Chemokines** | **6.195** | **5.575** | **0.620** | ***0.033*** |
| **B7H3** | **8.618** | **8.188** | **0.430** | ***0.057*** |
| **PD-L1** | **5.118** | **4.662** | **0.456** | ***0.057*** |
| **SOX2** | **4.357** | **3.701** | **0.656** | ***0.066*** |
| **IFNGamma** | **8.146** | **7.531** | **0.615** | ***0.068*** |
| **Stroma** | **7.618** | **7.032** | **0.586** | ***0.072*** |
| **PD-L2** | **4.179** | **3.631** | **0.548** | ***0.075*** |
| **Mammary.Stemness** | **5.284** | **4.287** | **0.997** | ***0.090*** |
| APM | 12.062 | 11.544 | 0.518 | 0.106 |
| Endothelial_Cells | 5.717 | 5.349 | 0.369 | 0.106 |
| Cell_Adhesion | 8.773 | 8.135 | 0.638 | 0.124 |
| CDK6_Expression | 6.079 | 5.751 | 0.328 | 0.161 |
| AR | 6.879 | 7.434 | -0.554 | 0.166 |
| Mast_Cells | 6.114 | 5.666 | 0.448 | 0.222 |
| Hypoxia | 7.110 | 6.891 | 0.219 | 0.226 |
| TGF_Beta | 9.121 | 8.831 | 0.290 | 0.228 |
| ER_Signaling | 11.701 | 12.037 | -0.336 | 0.263 |
| BC_Proliferation | 4.989 | 4.744 | 0.246 | 0.355 |
| PGR | 4.759 | 5.277 | -0.518 | 0.382 |
| ESR1 | 8.714 | 9.069 | -0.355 | 0.392 |
| BRCAness | 5.229 | 5.094 | 0.135 | 0.428 |
| PTEN | 6.817 | 6.927 | -0.110 | 0.452 |
| BC_p53 | 4.589 | 4.419 | 0.170 | 0.458 |
| FOXA1 | 9.246 | 9.431 | -0.185 | 0.461 |
| CDK4_Expression | 7.415 | 7.341 | 0.074 | 0.475 |
| MHC2 | 8.466 | 8.676 | -0.210 | 0.537 |
| Claudin_Low | 1.117 | 1.000 | 0.117 | 0.735 |
| ERBB2 | 7.907 | 7.969 | -0.062 | 0.806 |
| Rb1 | 7.076 | 7.064 | 0.012 | 0.916 |
| Apoptosis | 7.555 | 7.563 | -0.007 | 0.937 |

*p value using T test for independent variables considered significant less than 0.1 (marked in italics) for exploratory analysis.

**Supplementary Table 3.** Cox Survival univariate (PFS) analysis of the BC360^TM^ panel signatures in first line patients (n=31). PFS in months was chosen as the independent variable and high expression of each signature as the dependent variable of the cox model.

| BC360_Signature | Hazard Ratio | CI 95% | p value |
| --- | --- | --- | --- |
| **IFN.Gamma** | **7.195** | **1.986–26.072** | **0.003** |
| **Macrophages** | **4.340** | **0.970-19.424** | **0.055** |
| **IDO1** | **2.675** | **0.907-7.891** | **0.075** |
| **PD.L2** | **5.804** | **0.761-44.261** | **0.090** |
| **TIGIT** | **2.403** | **0.849-6.807** | **0.099** |
| SOX2 | 4.912 | 0.631-38.225 | 0.128 |
| Inflammatory.Chemokines | 3.132 | 0.704-13.933 | 0.134 |
| TIS | 2.360 | 0.747-7.458 | 0.144 |
| Treg | 1.918 | 0.655-5.620 | 0.235 |
| HRD | 1.735 | 0.614-4.903 | 0.298 |
| Differentiation | 1.583 | 0.560-4.472 | 0.386 |
| B7.H3 | 1.420 | 0.501-4.020 | 0.509 |
| Cytotoxic.Cells | 1.385 | 0.490-3.910 | 0.539 |
| PD.L1 | 1.431 | 0.545-4.509 | 0.541 |
| Cytotoxicity | 1.290 | 0.460-3.615 | 0.628 |
| PD.1 | 0.781 | 0.244-2.502 | 0.677 |
| Mammary.Stemness | 0.882 | 0.316-2.463 | 0.810 |
| Stroma | 1.073 | 0.378-3.046 | 0.895 |
| CD8.T.Cells | 1.019 | 0.368-2.819 | 0.972 |

*CI: confidence interval; p value using T test for independent variables considered significant less than 0.05 (marked in italics)
