# Supplementary material for: Immune‐based transcriptomic signature predicts CDK4/6 inhibitor efficacy in HR+/HER2– breast cancer
Source: Clin Transl Med. 2025 Aug 7;15(8):e70426. doi: 10.1002/ctm2.70426 (PMC12331873; doi:10.1002/ctm2.70426)

**SUPPLEMENTARY FIGURES LEGENDS**

**Supplementary Figure 1.** **Clinical characterization of the study cohort.** **(A)** Survival analysis of the cohort (n=100). Kaplan-Meier analysis of PFS (left) and OS (right). Data is expressed in months and the interquartile range (IQR). **(B)** Kaplan-Meier analysis of PFS according to distinct clinical characteristics associated to CDK4/6i treatment effectiveness. Patients are stratified according to treatment line, hormone sensitivity status, bone involvement and liver metastasis in first line patients (n=60). Hazard ratios (HR) and p-values were calculated using the log-rank test, with significance set at ≤0.05.

**Supplementary Figure 2. Tumor intrinsic subtypes impact survival of ABC patients treated with CDK4/6i. (A)** Distribution of intrinsic subtypes according to PAM50 in all evaluable tumors (n = 55), including primary (n = 31) and metastatic (n = 24) samples. Luminal A and B subtypes were predominant overall, while HER2-enriched and basal subtypes were more frequent in metastatic lesions (p = 0.034). **(B)** Kaplan–Meier analysis of progression-free survival (PFS) by intrinsic subtype among patients treated with CDK4/6i in the first-line setting (n = 31). Luminal A tumors tended to show longer PFS compared to HER2-enriched tumors, as previously reported. p-values were calculated using the log-rank test.

**Supplementary Figure 3.** **Cohort classifications in efficacy groups.** Schematic representation of the efficacy classification developed to dichotomize the cohort. The cutoff point to determine good or poor efficacy varies depending on the patient's treatment status. Patients were divided in three distinct groups: hormone-sensitive if *de novo* metastatic onset and no prior oncologic treatments or with relapses beyond 12 months after hormonal treatment; hormone-resistant if they relapsed during adjuvant hormonal treatment, within the first 12 months post-adjuvant treatment, or after first-line hormonal treatment as monotherapy or patients who had received chemotherapy for metastatic disease before CDK4/6i. For each group, the duration cutoff for good or bad efficacy was determined based on studies with similar clinical characteristics (represented in the figure). Of the 100 patients initially recruited, 91 patients had sufficient follow-up data at the time of the last analysis to be classified according to treatment response.

**Supplementary Figure 4.** **Differential gene expression and pathway analysis between efficacy groups and KIMA signature network.** **(A)** Heatmap representation of the 43 genes with significant (p < 0.05) differential expression between the good and bad efficacy groups in the BC360^TM^ cohort with sufficient follow-up (n = 47). Clusters were unsupervised using Pearson correlation**. (B)** Bar plots of the top 20 significantly downregulated (blue) and upregulated (red) genes in the poor response group compared to the good response group in the entire BC360^TM^ cohort, based on Log2 Fold Change (Log2FC) expression. **(C)** Gene Set Enrichment Analysis (GSEA) plot comparing good and bad efficacy groups from the BC360^TM^ cohort (n = 47), using Hallmark genesets. Transcriptional signatures comparing good and bad efficacy groups were generated using the normalized enrichment score (NES). Significance is represented as the adjusted p-value (FDR q-val).

**Supplementary Figure 5. Survival analysis according to the downregulated 5-gene set signature expression levels.** **(A)** Kaplan-Meier analysis of progression-free survival (PFS) and overall survival (OS) based on the downregulated gene expression levels in first-line patients (n = 31). (**B)** Scatter plot with mean±SEM comparing the mean expression of the 5-gene set signature between good and bad efficacy groups in all patients, regardless of the treatment line number (n=47). Significance was calculated using an unpaired t-test with Welch’s correction. ns, non-significative. **(C)** Kaplan-Meier analysis of PFS and OS based on the NEGIM signature expression in all patients (n = 47). p-values and hazard ratios (HR) in all plots were determined with the log-rank test. **(D)** Multivariate survival analysis of the BC360™ IFN-γ signature (high vs. low expression) adjusted for clinically relevant variables (Supplementary Figure 1B), including endocrine sensitivity (HS vs. HR), treatment line (1L vs. ≥2L), presence of liver metastases (Liver M1), and bone-only disease (Only Bone) in patients (n=47). HR, Hazard Ratio; CI, Confidence Interval.

**Supplementary Figure 6. Network map of KIMA signature.** Network map generated by IPA software. The graph illustrates the interactions between proteins from the KIMA signature (cyan), diseases (orange), and cellular functions (yellow) as implemented in the IPA database. The green node is associated with known biomarkers for breast cancer efficacy. White nodes represent intermediary proteins. Solid lines represent direct or robust interactions, while dotted lines represent indirect or less frequent interactions. Nodes are displayed in different shapes according to their type (proteins, diseases, cell functions, or biomarkers) and the type of protein (cytokine, enzyme, transcription regulator, transmembrane receptor, transporter, or group/complex).


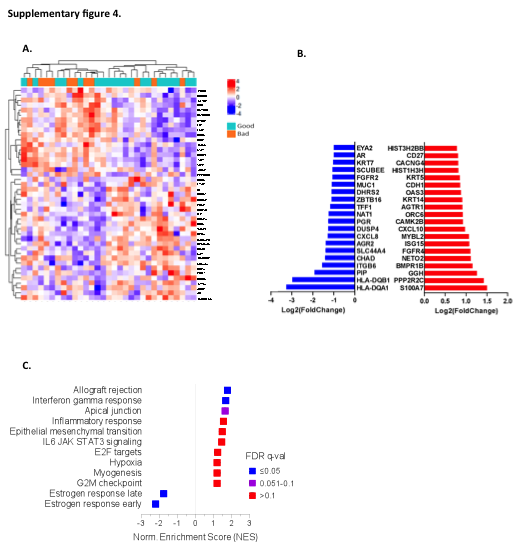

Supplement: Supplementary file 2 — Supporting Information [file CTM2-15-e70426-s003.docx]
