# Supplementary material for: Immune‐based transcriptomic signature predicts CDK4/6 inhibitor efficacy in HR+/HER2– breast cancer
Source: Clin Transl Med. 2025 Aug 7;15(8):e70426. doi: 10.1002/ctm2.70426 (PMC12331873; doi:10.1002/ctm2.70426)
Supplement: Supplementary file 3 — Supporting Information [file CTM2-15-e70426-s002.docx]

**Supplementary Information**

**Immune-Based Transcriptomic Signature Predicts CDK4/6 Inhibitor Efficacy in HR+/HER2– Breast Cancer**

Eudald Felip^1,2,3,4 ⴕ^, Edurne Garcia-Vidal^2,3 ⴕ^, Sara Cabrero-de las Heras^2,3,5^, Adrià Bernat-Peguera^2,4^, Beatriz Cirauqui^1,2,4^, Milana Bergamino^1,2,4^, Vanesa Quiroga^1,2,4^, Iris Teruel^1,2,4^, Angelica Ferrando-Díez^1,2,4^, Anna Pous^1,2,4^, Assumpció Lopez-Paradís^1,2,4^, Laia Boronat^1,2,4^, Marga Romeo^1,2,4^, Ricard Mesía^1,2,4^, Pedro Luis Fernandez^2,6^ Bonaventura Clotet^2,3,7^, Eva Riveira-Muñoz^2,3^, Anna Martínez-Cardús^1,2,4^*, Ester Ballana^2,3,7^*, Mireia Margelí^1,2,4^*

^1^ Institut Català d’Oncologia-Badalona, Hospital Universitari Gerans Trias i Pujol

^2^Translational Program in Cancer Research (CARE) Program in Health Research Institute Germans Trias i Pujol (IGTP), Hospital Universitari Germans Trias i Pujol, Universitat Autònoma de Barcelona, 08916 Badalona, Spain

^3^IrsiCaixa, Badalona, Barcelona, Spain.

^4^Badalona-Applied Research Group in Oncology (B-ARGO) in IGTP

^5^ Institute for Bioengineering of Catalonia (IBEC)

^6^ Department of Pathology, Hospital Germans Trias i Pujol, Badalona, Barcelona, Spain.

^7^Centro de Investigación Biomédica en Red de Enfermedades Infecciosas, CIBERINFEC

^ⴕ^ These authors contributed equally to this work.

*Correspondence: eballana@irsicaixa.es (E.B.); mmargeli@iconcologia.net (M.M.)

**MATERIALS AND METHODS**

**Clinical cohort and study design**

A prospective observational study was designed to include patients with ER+/HER2- ABC who started treatment with CDK4/6i in the Catalan Institute of Oncology (ICO) of Hospital Germans Trias i Pujol (HUGTIP), Badalona, Spain. Between May 2018 and April 2022, a total of 100 patients who started treatment with any of the three approved CDK4/6i (palbociclib, ribociclib, or abemaciclib) in any line of therapy for metastatic disease were enrolled, according to CDK4/6i approval status, or indications throughout the study period (2018-2022). The cohort's detailed clinical information and disease evolution were extracted from medical records (Table 1). Surplus 63 tumor blocks from clinical practice were collected for subsequent analysis in 55 patients, including paired samples from primary tumor and metastasis in 8 cases. All participants provided written informed consent specific to the study and tumor banking by the ethical protocols of Hospital Germans Trias i Pujol. The study was reviewed and approved by the Institution's ethical committee from the Hospital.

**Patient stratification based on clinical variables for functional analysis**

A clinical stratification approach for patients included was performed based on anticipated treatment efficacy, encompassing both hormone-sensitive and hormone-resistant populations of ABC, aimed at optimizing functional analysis and minimizing cohort heterogeneity. Different cut-off points were established for each situation, considering the diverse populations included and the progression-free survival (PFS) reported in the pivotal studies for the distinct CDK4/6i (1-6). This approach aimed to reduce the heterogeneity within the cohort and identify patients who derived maximum benefit from CDK4/6i, irrespective of treatment line or prior therapies (Supplementary Figure 1). The definition of each classification group was as follows:

Group 1: Hormone-sensitive patients with treatment-naive metastatic disease or a >12 months relapsing time after completing adjuvant endocrine therapy (ET). Categories: Good efficacy (PFS ≥ 24 months) vs. Bad efficacy (PFS < 24).

Group 2: Hormone-resistant patients with progression during adjuvant ET or within the first year after completion or after ≥ 1 line of hormone therapy (no previous chemotherapy). Categories: Good efficiency (PFS ≥ 12 months) vs. Bad efficiency (PFS < 12 months).

Group 3: Patients with prior chemotherapy for metastatic disease. Categories: Good efficiency (PFS ≥ 7 months) vs. Bad efficiency (PFS < 7 months).

Reanalysis of patient characteristics confirmed that the stratified groups were homogeneous in age and showed no significant differences in other clinical variables. This supports the robustness and simplicity of the dichotomous categorization for subsequent analyses (Supplementary Table 1).

**Tumor biopsies and RNA extraction**

Tumor samples were collected retrospectively, including primary tumors and metastatic biopsies obtained before the initiation of treatment. The Tumor Biobank and the Pathology Department of the Germans Trias i Pujol Hospital facilitated the selection and handling of these samples. For each sample, a pathologist identified areas enriched with tumor cells (minimum 40%) using hematoxylin-eosin-stained slides, ensuring the integrity of the selected regions for downstream analyses.

RNA extraction was performed from 6–10-micron tumor slices using the protocol established in the RNeasy FFPE Kit (Qiagen, Venlo, Netherlands). The quality of the RNA was determined using NanoDrop One (ThermoScientific) and Bioanalyzer 2100. Samples with a 260/280 ratio between 1.7 and 2.3 were included. RNA degradation was tested through the DV200 parameter, considering suitable samples with values over 40% for analysis. Samples that did not meet these requirements were discarded.

**Gene expression and bioinformatic analysis**

250ng of total RNA (50ng/µL) was used to analyze gene expression using the Breast Cancer 360™ (BC360^TM^) panel on the multiplexed digital nCounter® platform (NanoString Technologies, Inc., Seattle, WA, USA). BC360 ^TM^ panel includes 758 genes relevant to breast cancer, including those with established roles in tumor biology, the immune response, or the tumor microenvironment, and 18 housekeeping genes. Raw RCC files generated by the NanoString nCounter® platform were imported into nSolver™ Analysis Software (NanoString Technologies) for quality control, normalization to internal positive controls and housekeeping genes, and log2 transformation, following the manufacturer’s recommended protocols. For each sample, normalized data was used to determine correlation scores for the four Prosigna® intrinsic subtype signatures and assign intrinsic subtype according to published methods (NanoString Technologies, Inc.) (7, 8).

Gene Set Enrichment Analysis (GSEA) was performed using the expression of the 776 genes from the BC360 ^TM^ panel. Briefly, patients were stratified by efficacy group, and the mean expression for each gene was calculated within each group. Then, for each gene, the mean expression value of the good efficacy group was subtracted from that of the bad efficacy group. These differential expression values were then organized in decreasing order and used as a ranked input for a GSEA Pre-Ranked analysis with the Hallmark gene set database (h.all.v2024.1.Hs.symbols) from Molecular Signatures Database (MSigDB).(9-11).

Single gene expression analysis from first-line patients was used to select a putative predictive gene signature. Immune-related genes with at least a 50% significant expression (p-value ≤ 0.1) difference between the groups were selected, resulting in a list of 9 upregulated (*CXCL10, OAS3, STAT1, CD27, TIGIT, IL2RA, FOXP3, TAP1, TAP2*) and 5 downregulated (*HLA-DQA1, HLA-DQB1, CXCL8, TNFSF10, IL1B*) genes in the bad efficacy group. The protein interaction map of the selected upregulated genes was generated with QIAGEN IPA (QIAGEN Inc., https://digitalinsights.qiagen.com/IPA) (12). Intermediate proteins and connections between the nodes are the automated result of the Grow and Path Explorer tools using the IPA database. Diseases, cell functions and biomarkers nodes were manually added according to their relevance with our study scope and the number of connections with the signature members.

**Statistical Analysis**

Quantitative variables were represented using medians and ranges for clinical descriptive analysis, while qualitative variables were expressed with absolute frequencies and percentages. PFS was defined as the time (in months) from the initiation of treatment with CDK4/6i until treatment discontinuation due to disease progression or death from any cause. OS was the time (in months) calculated from the date of treatment initiation until death from any cause or the date of censoring at the last time the subject was known to be alive in an intention-to-treat population. Median PFS and OS were estimated using the Kaplan-Meier method and analyzed using the log-rank test, with statistical significance considered at a p-value < 0.05. The Kruskal-Wallis or Mann-Whitney U tests were applied for independent quantitative variables, and the chi-square test for qualitative variables, with a significance threshold of p-value < 0.05.

For survival analysis, Cox proportional hazards regression models were used to assess the prognostic and predictive relevance of intrinsic subtype and normalized gene/signature scores for PFS and OS. Hazard ratios (HR) and their associated 95% confidence intervals (CI) were reported. Scores relevant in univariate analyses (p<0.1) were further investigated through multiple logistic regression. Multivariate Cox regression models were used to evaluate the KIMA immune signature and IFN-γ as independent predictors of PFS, adjusted for relevant clinical variables. Thresholds for high/low expression were defined using ROC curves, and results were visualized using forest plots.

For the scatter plots representing the mean expression of gene signatures, each dot represents an individual patient. Data distribution for each plot was assessed with the Shapiro–Wilk test. When data was normally distributed, the significance was assessed by unpaired t-test analysis with Welch’s correction. Otherwise, the Mann-Whitney U test was performed.

The receiver operating characteristic (ROC) curves and area under the curve (AUC) values were calculated using the pROC package in RStudio (v4.5.1) to assess the predictive capacity of gene signatures. The optimal threshold value determined by this package was applied to classify patients into high or low-expression groups for each signature. Kaplan-Meier survival curves, scatter plots, GSEA plot and the representation of ROC curves were generated using GraphPad Prism (v.10.3.1), while RStudio was used for statistical analysis of clinical variables and for representing heatmaps (using the pHeatmap package) and volcano plots (using the ggplot2 package). We used Principal Component Analysis (PCA) to explore dimensionality reduction and potential correlation between the genes from the KIMA signature. The Elbow method was used to choose the number of principal components on the screen plot. For this analysis, the Jamovi project 2024 (v2.6) was used.

**References**

1. Finn RS, Martin M, Rugo HS, Jones S, Im SA, Gelmon K, et al. Palbociclib and Letrozole in Advanced Breast Cancer. N Engl J Med. 2016;375(20):1925-36.

2. Goetz MP, Toi M, Campone M, Sohn J, Paluch-Shimon S, Huober J, et al. MONARCH 3: Abemaciclib As Initial Therapy for Advanced Breast Cancer. J Clin Oncol. 2017;35(32):3638-46.

3. Slamon DJ, Neven P, Chia S, Fasching PA, De Laurentiis M, Im SA, et al. Phase III Randomized Study of Ribociclib and Fulvestrant in Hormone Receptor-Positive, Human Epidermal Growth Factor Receptor 2-Negative Advanced Breast Cancer: MONALEESA-3. J Clin Oncol. 2018;36(24):2465-72.

4. Sledge GW, Jr., Toi M, Neven P, Sohn J, Inoue K, Pivot X, et al. MONARCH 2: Abemaciclib in Combination With Fulvestrant in Women With HR+/HER2- Advanced Breast Cancer Who Had Progressed While Receiving Endocrine Therapy. J Clin Oncol. 2017;35(25):2875-84.

5. Cristofanilli M, Turner NC, Bondarenko I, Ro J, Im S-A, Masuda N, et al. Fulvestrant plus palbociclib versus fulvestrant plus placebo for treatment of hormone-receptor-positive, HER2-negative metastatic breast cancer that progressed on previous endocrine therapy (PALOMA-3): final analysis of the multicentre, double-blind, phase 3 randomised controlled trial. The Lancet Oncology. 2016;17(4):425-39.

6. Hortobagyi GN, Stemmer SM, Burris HA, Yap YS, Sonke GS, Paluch-Shimon S, et al. Updated results from MONALEESA-2, a phase III trial of first-line ribociclib plus letrozole versus placebo plus letrozole in hormone receptor-positive, HER2-negative advanced breast cancer. Ann Oncol. 2018;29(7):1541-7.

7. Sørlie T, Perou CM, Tibshirani R, Aas T, Geisler S, Johnsen H, et al. Gene expression patterns of breast carcinomas distinguish tumor subclasses with clinical implications. Proc Natl Acad Sci U S A. 2001;98(19):10869-74.

8. Prat A, Parker JS, Fan C, Perou CM. PAM50 assay and the three-gene model for identifying the major and clinically relevant molecular subtypes of breast cancer. Breast Cancer Research and Treatment. 2012;135(1):301-6.

9. Liberzon A, Birger C, Thorvaldsdóttir H, Ghandi M, Mesirov Jill P, Tamayo P. The Molecular Signatures Database Hallmark Gene Set Collection. Cell Systems. 2015;1(6):417-25.

10. Liberzon A, Subramanian A, Pinchback R, Thorvaldsdóttir H, Tamayo P, Mesirov JP. Molecular signatures database (MSigDB) 3.0. Bioinformatics. 2011;27(12):1739-40.

11. Subramanian A, Tamayo P, Mootha VK, Mukherjee S, Ebert BL, Gillette MA, et al. Gene set enrichment analysis: a knowledge-based approach for interpreting genome-wide expression profiles. Proc Natl Acad Sci U S A. 2005;102(43):15545-50.

12. Krämer A, Green J, Pollard J, Jr., Tugendreich S. Causal analysis approaches in Ingenuity Pathway Analysis. Bioinformatics. 2014;30(4):523-30.
